# Influence of ipilimumab on expanded tumour derived T cells from patients with metastatic melanoma

**DOI:** 10.18632/oncotarget.16003

**Published:** 2017-03-08

**Authors:** Jon Bjoern, Rikke Lyngaa, Rikke Andersen, Lisbet Hölmich Rosenkrantz, Sine Reker Hadrup, Marco Donia, Inge Marie Svane

**Affiliations:** ^1^ Center for Cancer Immune Therapy, Herlev Hospital, University of Copenhagen, Copenhagen, Denmark; ^2^ Department of Oncology, Herlev Hospital, University of Copenhagen, Copenhagen, Denmark; ^3^ Section for Immunology and Vaccinology, Technical University of Denmark, Copenhagen, Denmark; ^4^ Department of Plastic Surgery, Herlev Hospital, University of Copenhagen, Copenhagen, Denmark

**Keywords:** melanoma, tumour infiltrating lymphocyte, ipilimumab, CTLA-4, immunotherapy

## Abstract

**Introduction:**

Tumour infiltrating lymphocyte (TIL) based adoptive cell therapy (ACT) is a promising treatment for patients with advanced melanoma. Retrospective studies suggested an association between previous treatment with anti-CTLA-4 antibodies and long term survival after subsequent ACT. Thus, we hypothesized that treatment with anti-CTLA-4 antibodies can induce favourable changes to be detected in TILs.

**Results:**

Expanded T cells from Ipilimumab treated patients had a higher proportion of cells expressing CD27, intracellular CTLA-4, TIM-3 and LAG-3. In addition, broader and more frequent T cell responses against common tumour antigens were detected in patients treated with Ipilimumab as compared to anti-CTLA-4 naïve patients.

**Materials and methods:**

Expanded TILs were obtained from patients with advanced melanoma who had received Ipilimumab in the previous six months, or had not received any type of anti-CTLA-4 antibody. T cell specificity and expression of phenotypic and exhaustion markers were scrutinized as well as functional properties.

**Conclusions:**

Ipilimumab may induce tumor-infiltration of T cells of a more naïve phenotype expressing markers related to activation or exhaustion. Additionally, Ipilimumab may increase the frequency of T cells recognizing common tumour associated antigens.

## INTRODUCTION

Throughout the past decade, numerous new treatments for metastatic malignant melanoma have been tested and proven effective in clinical trials [1]. Several of these treatments take advantage of the immune system's ability to eradicate established tumour masses. Among these, one of the most promising experimental approaches is adoptive cell therapy (ACT), which has demonstrated efficacy in several independent clinical trials [2–5]. In this approach, tumour infiltrating lymphocytes (TILs) are first isolated from a metastasis from the individual patient. Thereafter, TILs are activated *in vitro* and massively expanded, and finally transferred back intravenously in combination with Interleukin (IL)-2 after pre-conditioning with lymphodepleting chemotherapy. Even though current ACT protocols have proven to be effective, safe and potentially curative treatments for metastatic melanoma, the majority of patients eventually experience tumour progression, clinical deterioration and death [6].

In order to increase the fraction of patients to benefit from this treatment, different factors could in principle be modulated, including, but not limited to, combining ACT with other treatments e.g. targeted therapies or immunomodulatory antibodies, with the aim of sensitizing the tumour cells or making the T cells more functionally competent. Interestingly, a retrospective analysis by Rosenberg et al. [6] suggested that prior immune checkpoint inhibition with recombinant anti CTLA-4 (Cytotoxic T Lymphocyte Antigen 4) antibody, followed by progression and thus infusion of TILs, was associated with a markedly high five year survival. Several rationale explanations of this phenomenon could be suggested. Thus, it is possible that anti-CTLA-4 treatment genuinely increases the response to ACT. However, the survival data could also be an artefact due to reduced biological aggressiveness of disease in patients fit to receive both anti-CTLA-4 antibody treatment and subsequent ACT. Therapeutic antibodies targeting CTLA-4 have been widely tested in clinical trials [7]. Ipilimumab, an IgG1 blocking CTLA-4 signaling, was approved for the treatment of metastatic melanoma in 2011. This antibody works through blockade of an early immune checkpoint on T cells, which promotes APC-mediated T cell activation and thereby increase T cell specific immunity including antitumor immune responses [8]. It is also suggested that a contributing (if not essential) mechanism is elimination of regulatory T cells (Tregs) [9]. In this study, we provide mechanistic insight as to how pre-treatment with Ipilimumab may induce measurable phenotypic and functional changes of TILs, which may in turn explain the increased survival of melanoma patients treated with TIL-based ACT who were previously treated with Ipilimumab.

## RESULTS

### Patients

Tumour samples were collected prospectively as part of standard-of-care surgery or after enrollment in a clinical trial. A total of 34 patients were included in the analysis; 15 Ipilimumab naïve and 19 treated within 6 months prior to tumour removal. Table 1 summarizes patient characteristics. As seen, the Ipilimumab naïve patients were on average ten years older and had received less systemic treatments than the Ipilimumab treated patients.

**Table 1:** Patient demographics

	Ipilimumab naïve	Ipilimumab within 6 months
Number of patients	15	19
Mean age at resection (SD)	56.8 (10)	50.3 (13)
No. Ipilimumab treatments		
2	0	2
3	0	1
4	0	16
Number of systemic treatments (SD)	0.5 (0.7)	1.9 (1)
Temozolomide	1	1
Vemurafenib	0	2
IL2	5	13
ACT	1	0
DC vaccination	0	1
Ipilimumab	0	19
Disease stage		
IIIc	1	1
M1a	2	3
M1b	1	0
M1c	11	15
Trial		
Clinical trial	11	19
Research protocol	4	0
Gender: male/female	5/10	9/10

Patient demographics at the time of tumour removal for T cell culture. Abbreviations: SD (standard deviation) IL2 (interleukin 2) ACT (adoptive cell therapy) DC (dendritic cell).

### Generation and composition of young TILs

**Figure 1:**
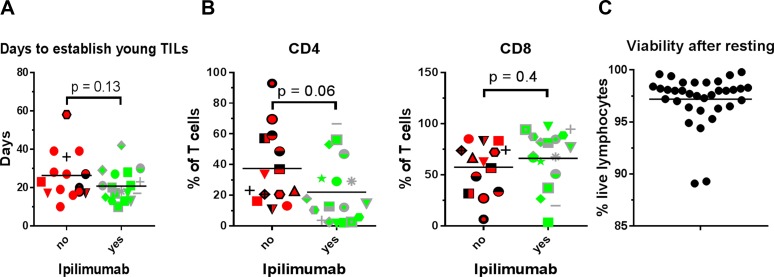
Young TIL generation, CD4/CD8 phenotype and cell viability (**A**) Days to establish young TIL i.e. duration from initiation of T cell culture until the total cell count reaches > 50 × 10^6^ sorted according to whether the patient had received Ipilimumab prior to tumour removal. (**B**) Percentage of T cells expressing CD4 or CD8 in the expanded TILs sorted according to whether the patient had received Ipilimumab prior to tumour removal. (**C**) Proportion of lymphocytes not staining positive for the dead cell marker Near Infra-Red, after resting of expanded TILs in IL2 free media.

TIL expansion is accomplished by culturing of small tumour fragments in the presence of IL2, and young TILs are defined as pooled cultures of > 5 × 10^7^ total cells originating from approximately 24 fragments. Time used for this process varies and could theoretically be influenced by several factors. As shown in Figure 1A, the duration of time to establish young TILs is not affected by prior treatment with Ipilimumab. We performed flow cytometric analysis in order to assess whether prior treatment with Ipilimumab could affect the frequency of CD4^+^ and CD8^+^ T cells. Figure 1B depicts the frequency of CD4^+^ and CD8^+^ T cells. As seen, the distribution of these cell subsets varies among patients, with a tendency towards a lower frequency of CD4^+^ cells in the Ipilimumab treated group compared to the Ipilimumab naïve though the difference was not significant.

### Phenotype and exhaustion profile

**Figure 2:**
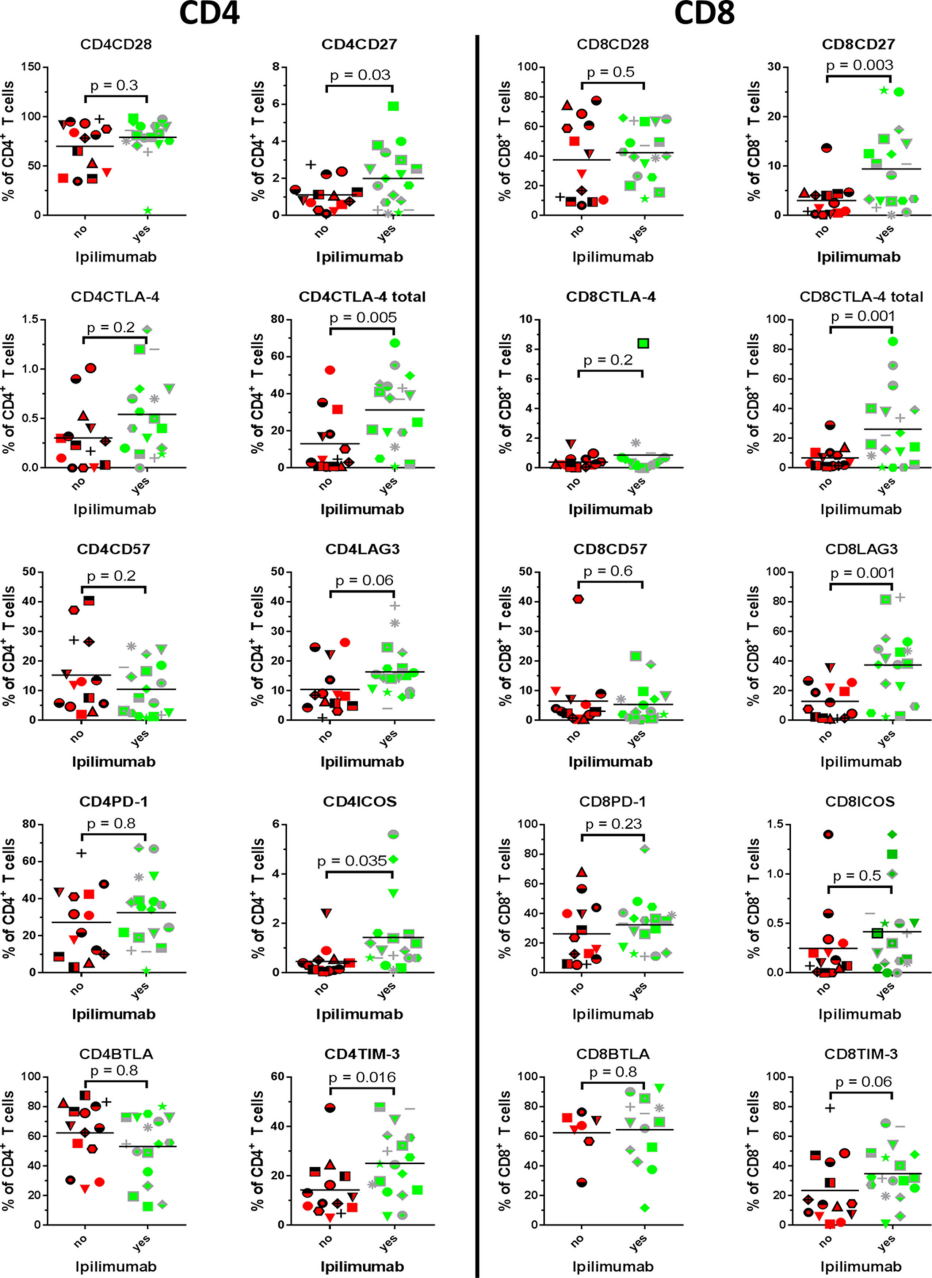
Phenotype- and exhaustion markers Proportion of expanded tumour infiltrating T cells expressing the depicted markers were compared between patients treated with Ipilimumab before removal of tumour and patients naïve to anti-CTLA-4 treatment.

T cells display a vast repertoire of different surface markers that may reflect the functionality of the cell [10]. We performed phenotypic analysis on the expanded TILs in order to assess whether any markers were consistently affected in CD4^+^ and CD8^+^ T cell subsets, depending on prior Ipilimumab treatment. The expression of selected markers may be quantitatively affected by culture conditions, most importantly the concentration of IL2 which may transiently suppress e.g. CD27 expression [11]. Therefore, cells were rested for two days in IL2-free media before analysis. Viability of expanded TILs after this resting-step was generally high (Figure 1C). We assessed the expression of a number of different receptors implicated in T cell activation and regulation. Results are presented in Figure 2. In general, expanded CD4^+^ and CD8^+^ T cells displayed a phenotype compatible with an activated and/or exhausted phenotype, with expression of programmed death receptor 1 (PD-1), B- and T-lymphocyte attenuator (BTLA), CD57 and CD28.

### ICOS

The CD28 super family activation marker inducible T cell co-stimulator (ICOS) has been linked to a better outcome of treatment with Ipilimumab if up-regulated during treatment [12]. As seen in Figure 2, the proportion of cells expressing ICOS were only modestly higher in both CD4^+^ T cells (median 0.35% Ipilimumab naïve vs. 0.9% Ipilimumab treated) and CD8^+^ T cells (median 0.12% Ipilimumab naïve vs. 0.35% Ipilimumab treated) from patients that had been treated with Ipilimumab, and the difference was statistically significant only when comparing CD4^+^ T cell subsets (*p* = 0.035 for CD4^+^ T and *p* = 0.5 for CD8^+^ T).

### CD27

CD27 is expressed on T cells giving rise to memory responses [13], and expression of CD27 in T cells used for ACT confers a higher likelihood of a clinical response [6]. As seen, both CD8^+^ and CD4^+^ T cells from patients that had received Ipilimumab uniformly demonstrated higher frequencies of CD27^+^ cells (*p* = 0.03 for CD4^+^ and *p* = 0.003 for CD8^+^). Expression was in general absent or diminutive in CD8^+^ T cells from Ipilimumab naïve patients, whereas a small proportion of CD4^+^ T cells displayed expression. In general, CD8^+^ T cells had higher frequencies of CD27+ cells, compared to CD4^+^ T cells.

### CTLA-4

CTLA-4 is an important regulator of T cell function and reactivity, especially during priming of immune responses [14]. Ipilimumab targets CTLA-4 and is likely to have effect on the dynamics of this molecule. We analyzed the level of expression on the surface and total expression (surface + intracellular) of CTLA-4. As seen from Figure 2 (2nd line from the top), the surface-expression of CTLA-4 is generally low in both CD4^+^ T cells and CD8^+^ T cells. There was a trend towards a higher surface expression in CD4^+^ cells from Ipilimumab treated patients, however non-significantly (*p* = 0.2). When comparing total expression of CTLA-4, i.e. the positive fraction in permeabilized cells, in Ipilimumab naïve and treated, we found uniformly higher expression in both CD4^+^ and CD8^+^ cells from patients treated with Ipilimumab (*p* = 0.005 and *p* = 0.02, respectively).

### TIM-3

TIM-3 is an immune inhibitory molecule first identified as a regulator of Th1 cells [15] and implicated in T cell dysfunction in chronic HIV infection [16] and in TILs from melanoma patients [17]. There was a significant trend towards higher proportion of CD4^+^ T cells expressing TIM-3 (*p* = 0.016) in Ipilimumab treated patients. In CD8^+^ T cells, expression of this antigen was higher in patients treated with Ipilimumab compared to untreated, however not statistically significant (median 14.3% vs. 31.8%, Mann-Whitney test *p* = 0.06).

### LAG-3

Lymphocyte-activation gene 3 (LAG-3) is up-regulated on T cells upon activation, and functions as a regulatory protein, preventing signaling through CD3, when interacting with its receptor HLA class II [18]. In a murine model, LAG-3 has been shown to mediate tumour immune-escape through tolerance [19], and synchronous knock-out of LAG-3 and PD-1 confers resistance to tumour xenografts [20]. We found a significantly higher median expression of LAG-3 in CD8^+^ T cells from patients treated with Ipilimumab compared to Ipilimumab naïve, mean 9.9% vs. 37.95%, (student *T* test *p* = 0.001). We found no significant difference in LAG-3 expression in CD4^+^ T cells (*p* = 0.06).

### CD8+ and CD4^+^ T cell anti-tumour reactivity

**Figure 3:**
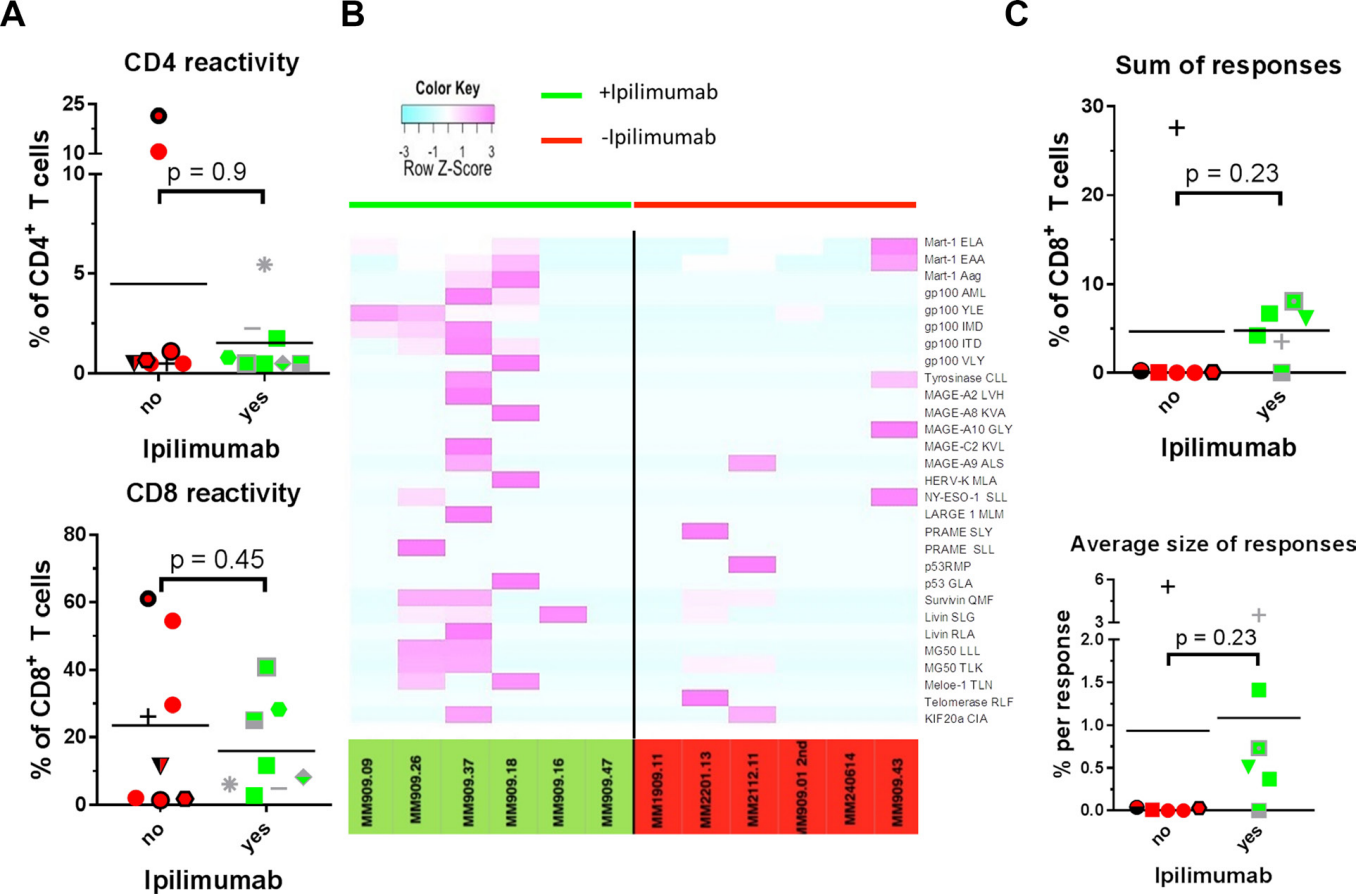
T cell reactivity and specificity Reactivity of expanded tumour infiltrating T cells was tested in a co-culture assay with autologous cancer cells and assessed with intracellular cytokine staining. T cell specificity was measured using combi coding with MHC tetramers loaded with a panel of tumour antigen derived peptides in HLA-A2 ^+^ patients. Patients treated with Ipilimumab were compared with patients naïve to anti-CTLA-4 treatment. (**A**) T cell reactivity against autologous cancer cells in CD4^+^ (top) and CD8^+^ (bottom) T cells. (**B**) Heat map representing row Z-score for HLA-A2 restricted T cell responses found in 12 HLA-A2^+^ patients. Patients left of the black line had received Ipilimumab prior to removal of tumour whereas patients depicted on the right of the line were anti-CTLA-4 treatment naïve. (**C**) Top: sum of frequencies of T cells with specificity against common tumour antigens. Bottom: average size of a T cell response.

Our group recently reported that T cell reactivity towards autologous and allogeneic melanoma cell lines is positively correlated to treatment response in TIL based ACT [5], that Ipilimumab has been shown to induce influx of T cells in tumour lesions in patients with clinical benefit [22] and might have an impact on T cell reactivity towards tumour cells. T cell reactivity in terms of cytokine and CD107a production upon co-culture with autologous cancer cell lines using intracellular cytokine staining and flow cytometry analysis was assessed. Cancer cells were treated with IFN-γ before co-culture in order to up-regulate antigen presenting machinery [21]. T cells were deemed tumour reactive when positive for either CD107a, IFN-γ or TNF-α or a combination of these (see methods section). Though the median level of reactivity in CD8+ T seemed slightly lower in TILs from Ipilimumab treated patients (*p* = 0.5), the sample size and level of difference does not allow firm conclusions to be drawn in terms of reactivity against autologous tumour cells. Results are presented in Figure 3A.

### Antigen specificity

We performed combinatorial coding with MHC class I tetramers and flow cytometric analysis in order to assess the frequencies of specific melanoma-associated antigen-specific cells among the TILs [23]. Samples were screened for specificity against a panel of 175 HLA-A2 restricted melanoma associated T cell epitopes from published tumour associated antigen [24]. Prior to analysis, patient samples were HLA typed, and antigen specificity was assessed with HLA-matched tetramers. Only HLA-A2+ patients were included in these analyses, and only epitopes towards which at least one patient harbor immune responses are included in the figures. Results are presented in Figure 3B–3C and Table 2. In general, we found frequent responses towards shared tumour antigens. The heatmap in Figure 3B illustrates that responses were generally broader and more frequent in Ipilimumab treated patients as evident by purple colours. As seen in Figure 3C, a tendency for higher frequency of responses towards common tumour antigens was detected in Ipilimumab treated patients, and the average size of a response tended to be higher after Ipilimumab treatment. The differences between the two groups for both parameters were not statistically significant (*p* = 0.2 for both), but the sample size in these experiments is not suitable for formal hypothesis testing.

**Table 2:** T cell specificity of expanded tumour infiltrating T cells

Antigen type	Patient	MM.909.09	MM.909.26	MM.909.37	MM.909.18	MM.909.16	MM.909.47	MM.1909.11	MM.2201.13	MM.2112.11	MM.909.01 2nd	MM.2406.14	MM.909.43
	Antigen												
Differentiation	Mart-1 ELA	1.7	0.03	0.4	3.4					0.01	0.03		15.6
	Mart-1 EAA		0.04	0.6	2.9				0.003	0.006			4.1
	Mart-1 Aag			0.1	1.5								
	gp100 AML			0.008	0.008								
	gp100 YLE	2.5	1.8	0.03	0.03						0.03		
	gp100 IMD	0.022	0.1	0.4									
	gp100 ITD		0.005	0.3	0.01								
	gp100 VLY				0.04								
	Tyrosinase CLL			0.8									0.3
Cancer Testis	MAGE-A2 LVH			0.004									
	MAGE-A8 KVA				0.03								
	MAGE-A10 GLY												1.9
	MAGE-C2 KVL			0.1									
	MAGE-A9 ALS			0.005						0.006			
	HERV-K MLA				0.04								
	NY-ESO-1 SLL		0.3										5.7
	LARGE 1 MLM			0.1									
Over Expressed	PRAME SLY								0.03				
	PRAME SLL		1.1										
	p53RMP									0.005			
	p53 GLA				0.08								
	Survivin QMF		0.3	0.3					0.02	0.009			
	Livin SLG		0.3	0.5		3.5			0.06				
	Livin RLA			0.006									
	MG50 LLL		0.04	0.04					0.03				
	MG50 TLK		2.1	2.2						0.001			
	Meloe-1 TLN		0.01		0.03								
	Telomerase RLF								0.1				
	KIF20a CIA			0.006						0.004			

T cell specificity of expanded tumour infiltrating T cells assessed with combi coding. Green background indicates patients had received Ipilimumab up to six months before tumour removal. Red background indicates Ipilimumab naïve patients. Numbers indicate the percentage of CD8 T cells staining positive for a MHC/peptide tetramer loaded with the given peptide antigen.

## DISCUSSION

In this work, we investigated whether Ipilimumab induces measurable changes on phenotype, antigen specificity, and functionality of (expanded) tumour-infiltrating T cells. Samples were collected prospectively from patients receiving standard of care surgical resection of tumor metastases (mostly for palliative reasons) or for enrolment in ACT trials.

In general, we found high expression of markers traditionally regarded as immune inhibitory molecules including PD-1, BTLA, LAG-3 and TIM-3. Additionally, a high proportion of both CD4^+^ and CD8^+^ T cells expressed CD28, associated with an early or intermediate phenotype of the TILs [25]. To our knowledge, it is not well established to what extent the culture process used for generating T cells for current ACT protocols affects the phenotype or functionality of TILs, though we recently reported that the relative distribution of T cells expressing known antitumor functions seems maintained at stable levels [26]. Several groups have, however, reported that the tumour microenvironment induces T cell unresponsiveness towards tumour cells, which is apparent by high T cell expression of inhibitory markers [17], [20], [27], which could point to a similarity between the phenotype of tumour infiltrating lymphocytes *in vivo* and after expansion *in vitro*. On the other hand, the rapid expansion protocol includes culture conditions delivering stimuli far exceeding physiologic activation, which might induce homeostatic expression of inhibitory molecules on its own.

We found that several phenotypic markers were affected by administration of Ipilimumab prior to surgical removal of tumor samples used to isolate and expand TILs, suggesting that Ipilimumab induced marked changes in T cell infiltrates, which can still be detected despite heavy *in vitro* expansion. This is particularly striking, as Ipilimumab was administered relatively short before tumor removal and the *in vitro* process involved several rounds of expansion, which may presumably take several months or years in a comparable *in vivo* physiological setting. Of note, clinical responses induced by Ipilimumab may last for over a decade [28], thus it is not surprising that measurable changes in immune parameters can be detected despite harsh manipulation.

Surface expression of the inhibitory receptor CTLA-4, which is targeted and neutralized by Ipilimumab, were generally absent on T cells from both groups of patients. However, upon permeabilization of the cell membrane our analysis revealed high intracellular expression of this antigen and Ipilimumab treatment appeared to be associated with higher frequencies of CTLA-4^+^ cells in both the helper and cytotoxic T cell compartments. The implication of this is not known, but may reflect a physiologic counter regulation upon treatment with anti-CTLA-4 antibody. Interestingly, higher pre-treatment levels of intracellular CTLA-4 has been associated with a better overall survival in prostate cancer patients treated with Ipilimumab [29]. Several articles have reported ICOS up-regulation during treatment with Ipilimumab, as a strong correlate with subsequent clinical response [29], [30]. Up-regulation of this activation marker may be evident long after treatment discontinuation [31], and a recent publication has indicated that the ICOS^+^ T cells may be the actual effector cells conveying the anti-tumour effect of Ipilimumab in a murine model [32]. We scrutinized whether Ipilimumab affected surface expression of ICOS on T cells. Indeed, we found only a very low fraction expressing ICOS with a slightly higher expression in CD4^+^ T cells in the Ipilimumab treated group. No significant difference was demonstrated in CD8^+^ T cells, which is in accordance with a more pronounced effect of Ipilimumab on ICOS expression on the CD4^+^ T cell compartment [31]. Furthermore, expression of TIM-3 and LAG-3 were significantly higher in CD8^+^ T cells from patients treated with Ipilimumab, and with regard to the former, borderline significantly higher in CD4^+^ T cells. LAG-3 is an inhibitory molecule with structural similarity to CD4, which, upon binding by its ligand MHC class II, associates with the CD3/T cell receptor (TCR) complex and reduces signal-strength through the TCR thereby inhibiting proliferation and cytokine response [18, 33]. It has been implicated as an important mediator of tumour immune escape in melanoma [34], and a neutralizing antibody developed by Bristol-Myers Squibb is currently being tested in combination with Nivolumab (clinicaltrials.gov identifier NCT01968109). TIM-3 also functions as an immune inhibitory receptor specifically expressed on Th1 and Tc1 inflammatory cells [15], which may induce cell death when engaging its ligand galectin-9 [35]. However, signaling through this receptor is highly complex, and cells may be salvaged from galectin-9/TIM-3 induced apoptosis by expression of the intracellular signal-repressor Human Leucocyte Antigen B-associated transcript 3 [36], making interpretation of TIM-3 expression ambiguous. The underlying mechanism for the observed up-regulation of LAG-3 and TIM-3 is not clear, though it could be speculated that Ipilimumab, by blocking the effect of an important regulatory molecule on T cells, induces counter regulatory homeostatic mechanisms. Another possibility is that Ipilimumab attracts highly activated T cells to the tumour, which are likely to express a variety of both regulatory and inflammatory markers.

Response towards autologous tumour cells displayed a trend towards lower reactivity in Ipilimumab treated patients, though not significantly. However, Ipilimumab induces an influx of cells with a more naïve phenotype into the tumour microenvironment [32], which may not be capable of producing cytokines without further differentiation [10] and can be especially evident in short-term *in vitro* assays providing temporary limited stimuli. Despite this, the cells may in fact be tumour specific and reactive after activation and proliferation. In accordance with this, we found a markedly higher proportion of both CD4^+^ and CD8^+^ T cells expressing CD27, which is associated with a more naïve phenotype and longer telomere length [6]. CD27 expression was generally very low or even absent when analyzing cells right after rapid expansion, where cells have been exposed to very high concentrations of IL2. We thus confirm data from Huang et al. [11] demonstrating that a subset of T cells will re-express CD27, and possibly other markers, when withdrawn from supra-physiologic cytokine concentration. The higher proportion of CD27^+^ T cells in Ipilimumab treated is notable for several reasons; it indicates that Ipilimumab has long lasting impact on T cell dynamics, which will be evident even after extended culture and expansion. Additionally, this marker is associated with a higher chance of engraftment upon transfer into patients, which, in turn, is associated with better clinical outcome of ACT [6]. Taking advantage of the combinatorial coding technique, we screened expanded cells for reactivity towards a library of epitopes from shared tumour associated antigens. In order to compare data between patients, only HLA-A2 positive patients were included in the analyses. The analyses suggest that responses were less frequent and had specificity against fewer different epitopes in Ipilimumab naïve patients. This finding may suggest that a higher fraction of adoptively transferred T cells is genuinely tumour specific, but this was not directly supported by functional data on reactivity against autologous cancer cell lines. However, cancer cells used in these assays may not fully represent the whole complexity and heterogeneity of an *in vivo* growing tumor. Other factors, including antigen loss or down-regulation of antigen processing and presentation machinery may influence the antitumor reactivity, as well as a more naïve phenotype may influence the frequency of true tumor-specific cells able to generate measurable immune responses in short-term assays. This means that our functional assays may actually underestimate the proportion of tumour specific T cells depending on the individual characteristics of the autologous tumor cell line.

Interestingly, Kvistborg et al. [37] found broadened, but not increased, magnitude of tumor-antigen specific T cell responses in the peripheral blood of patients treated with Ipilimumab.

The epitope-library used in our specificity screening is vastly derived from shared tumour associated antigens, and hence a significant fraction of genuine tumour specific T cells may be missed. It has previously been shown that TILs may be neoantigen-specific [38, 39], and previous work from our group has demonstrated that only a small fraction of the expanded T cells can be accounted for in terms of specificity [24]. Despite this, our findings still indicate that responses towards tumour associated antigens were more frequent and broader in Ipilimumab treated patients.

It should be kept in mind that patients in the Ipilimumab group were referred to surgical tumour resection and ultimately ACT because they lacked disease control after check point inhibition. Consequently our findings do not necessarily reflect TIL phenotype and tumour reactivity in clinical responders to Ipilimumab. However, these patients do indeed represent relevant candidates for TIL based ACT. In conclusion, we have identified several T cell-related factors impacted by prior treatment with Ipilimumab. Especially the higher expression of CD27, which has been linked to better outcome of ACT, supports a beneficial effect of Ipilimumab before harvest and expansion of tumour-infiltrating lymphocytes. A phase I clinical study (clinicaltrials.gov identifier NCT01988077) conducted at the Sheba Medical Center, Israel, is currently assessing response rate and evaluating toxicity in a trial combining Ipilimumab with ACT. This study will give indications as to whether the combination is appropriate in a prospective trial. Outcome in such a trial may be even better than estimated from the retrospective analysis published by Rosenberg et al [6], given that patients treated with Ipilimumab in our study were selected for ACT because they did not experience disease control with Ipilimumab.

## MATERIALS AND METHODS

### Patients and tumour specimens

All procedures were approved by the Scientific Ethics Committee for the Capital Region of Denmark. Written informed consent was obtained from patients before any procedure according to the Declaration of Helsinki. Metastatic tumour specimens of at least 1cm^3^ were obtained from patients with melanoma stage IV undergoing standard-of-care surgical procedures as part of a research protocol approved by the local ethics committee (approval no. H-2-2014-052) or from specimen collection for enrollment in a clinical trial (Clinicaltrials.gov Identifier: NCT 00937625). Only patients either treated with Ipilimumab within six months prior to tumour removal or patients that were anti-CTLA-4 naïve were included in the study. As a consequence of this study design, patients in the Ipilimumab group were selected based on treatment failure to Ipilimumab. Part of the samples used in this publication, have also been used for analyses in previously published studies [5, 21, 24, 40–41].

Fresh specimens were immediately transported to the laboratory in cell media (RPMI 1640). The tumour masses were isolated from the surrounding tissues, and tumours were sliced into multiple fragments (1–3 mm^3^ each) with a scalpel.

### Reagents and cells for TIL and autologous tumour generation

Human AB serum (HS) was purchased from Sigma- Aldrich (Brøndby, Denmark); RPMI-1640 with GlutaMAX and AIM-V medium were obtained from Invitrogen (Nærum, Denmark). RhIL2 (Proleukin) was from Novartis (Basel, Switzerland), and OKT3 (anti-CD3) antibody was from Cilag AG (Schaffausen, Switzerland). Pulmozyme was purchased from Roche (Basel, Switzerland). Allogeneic peripheral blood mononuclear cells (PBMCs) (or feeder cells) were obtained from buffy coats from healthy donors. Solu-Cortef (hydrocortisone sodium succinate) was obtained from the local hospital pharmacy. Fetal bovine serum (FBS) was from Gibco (Nærum, Denmark). Complete medium (CM) consisted of RPMI-1640 with GlutaMAX, 25 mM HEPES pH 7.2, 100 U/ml penicillin, 100 μg/ml streptomycin and Fungizone^®^ (Bristol-Myers Squibb, New York, NY, USA) 1,25 lg/ml supplemented with 10% HS and 6000 IU/ml of rhIL2. Rapid expansion medium (RM) consisted of AIM-V medium and Fungizone^®^ 1,25 lg/ml supplemented with 6000 IU/ml rhIL2.

### TIL generation and melanoma culture generation

Young TIL cultures were generated from tumour fragments cultured in IL2-containing culture medium, as previously described [42–44]. Briefly, on day 0, individual tumour fragments were placed in the wells of a 24-well plate with 2 ml of CM per well. Cell cultures were kept in a humidified atmosphere at 37°C, with 5% CO_2_ in air. Cell density and morphology were assessed every other day. Within one week after initiation of the culture, half of the culture media was carefully aspirated and replaced with fresh. This was subsequently repeated three times per week. Young TILs were defined as pooled cultures of at least 5 × 10^6^ total cells originating from approximately 24 fragments that had expanded to confluent growth of the original 2-ml wells and eliminated adherent tumour cells. The definition of young TILs is based on the minimum cell number that would be needed for therapeutic protocols according to previously published studies [2, 43–47] and our internal experience. Autologous melanoma cultures were established from additional tumour fragments or from transport media from the same metastatic lesions, and cultured in RPMI 1640 supplemented with 10% FBS and 500 ng/ml of Solu-Cortef for inhibition of lymphocyte proliferation.

### Rapid expansion protocol

Cryopreserved or freshly prepared TILs were expanded in a 14-day Rapid Expansion Protocol (REP). Briefly, cultures were initiated in upright T175 flasks (Nunclon, 1 × 10^6^ TILs) or T25 flasks (1 × 10^5^ TILs) in standard culture medium supplemented with stimulating anti-CD3 antibody, allogeneic gamma irradiated peripheral blood mononuclear feeder cells in a ratio of 200:1 TILs and high doses of interleukin-2, as described previously [40]. On day 7, TIL cultures were either continued in the Wave bioreactor system, as previously described [48], or in static conditions [40]. Cells were allowed to proliferate for a total of 14 days according to the REP, after which cells were cryo-preserved in 90 % human AB serum with 10% dimethyl sulphoxide (Herlev Hospital Pharmacy) until further analysis.

### Exhaustion profile analysis

Cells were thawed and cultured in AIM-V culture medium with 5% heat-inactivated human serum in 24-well plates at a concentration of 3–5 × 10^6^ cells/ml. Plates were incubated in a humidified incubator at 37°C, with 5% CO_2_. After 48 hours of resting, cells were harvested and washed in FACS buffer before surface staining. For exhaustion profile analysis, cells were stained at 4°C for 30 min., washed, and, for tubes only intended for surface staining, resuspended in FACS buffer. For tubes intended for intracellular staining, cells were permeabilized using BD Bioscience Cytofix/CytopermTM Kit according to manufacturer's instructions. The following antibodies were used: CD4-PerCP, CD57-FITC, CD27-PE, CD56-PE-Cy7, CD28-APC, CD8-AmCyan, ICOS-PE, BTLA-PE, CTLA-4-APC, PD-1-PE-Cy7 (all from BD Bioscience, San Jose, CA, USA), Near Infra Red dead cell marker (Dako), LAG-3-FITC (LifeSpan Biosciences), TIM-3 (eBioscience), CD107a; PECy7-conjugated IFN-c; PerCP-conjugated CD8; APC-conjugated CD4, TNF-a; APCCy7-conjugated CD3. Cells were resuspended in FACS buffer prior to acquisition, and the cells were acquired using a BD FACSCanto II flow cytometer. A minimum of 100 000 events were recorded per sample. Analysis was performed with the BD FACSDiva Software (BD Bioscience,)

### Anti-tumour activity of TILs and FACS analysis of melanoma cells

For simultaneous CD107a and intracellular cytokine analysis, TILs were cultured for 5 hours at 37°C with 5% CO_2_ in air in the presence or absence (negative control) of autologous cancer cells at an effector/target (E:T) ratio of 3:1, as previously described [40]. Autologous cancer cells used as target cells were pretreated with 100 IU/ml interferon (IFN) g for 72 hours. Cells were acquired using a BD FACSCanto II flow cytometer (BD, Albertslund, Denmark). At least 200,000 live TILs were acquired. Analysis was performed with the BD FACSDiva Software. Criteria to define a positive anti-tumour response were as follows: a frequency of CD107a or cytokine positive TILs of at least twice the frequency of the background and at least 50 positive events; otherwise, at least 10 times the background. Cells were deemed tumour reactive when positive for either CD107a, IFN-g or tumour necrosis factor (TNF) α or a combination of these.

### Major histocompatibility complex (MHC) multimer-based analyses of antigen specific T cells

The generation of combinatorial encoded MHC multimers and the gating strategy is described in detail by Hadrup et al. [49, 23]. MHC multimers were produced using conditional ligand exchange technology, for the HLA ligands HLA-A1, A2, A3, A11 and B7 [50, 51]. For detection of antigen-specific T cell populations we made use of combinatorial encoding with MHC multimers, in brief, peptide-MHC monomers were multimerized with two different fluorescence-labeled streptavidin conjugates for each peptide specificity, enabling the simultaneous testing of 28 different specificities in a single sample by combining 8 colors into dual-color codes for MHC multimers, in order to measure specific T cell populations with flow cytometry. The cells were additionally stained with LIVE/DEAD® Fixable Near-IR Dead Cell Stain Kit for 635 nm excitation (Invitrogen, Life Technologies, Naerum, Denmark), CD8-Alexa Flour 700 (BD Pharmingen, Albertslund, Denmark) or CD8-peridinin chlorophyll (PerCP) (Invitrogen, Life Technologies, Naerum, Denmark) and fluorescein isothiocyanate (FITC) coupled antibodies to CD3 or to a panel of CD4, CD14, CD16, CD19 (all from BD Pharmingen, Albertslund, Denmark) and CD40 (AbD Serotec, Puchheim, Germany). Data acquisition was performed on an LSR II flow cytometer (Becton Dickinson) with FacsDiva software. Positive responses were defined by a minimum of 10 events at a frequency of minimum 0.002% of CD8 T cells. We used streptavidin conjugated fluorochromes: Qdot- 585(Q10111MD), 605 (Q10101MD), 625 (A10196), 655 (Q10121MD), 705 (Q10161MD) all from Invitrogen and PE-Cy7 (405206), APC (405207) and PE (405203) from Biolegend to encode the specific MHC multimers. MHC multimers were prepared at 100ug/ml and cells were stained using 2ul of each double colored MHC multimer. First, we gated on lymphocytes followed by single cells, live cells, and CD8 cells. Gates were made for each single MHC multimer color and then combined to gate out events positive for only one MHC multimer color and three or more MHC multimer colors, while showing events positive for exactly two MHC multimer colors.

### Statistics

Box plots show the median (horizontal line) inside a box spanning the 25th to the 75th percentile. Whiskers extend 1.5 times the interquartile range or, if no case has a value in that range, to the minimum or maximum values. A horizontal line in dot plots represents the median. Data were analyzed using SPSS software (Version 19.0, IBM Cooperation, Somer, NY, USA). Graphs were made using SPSS software or GraphPad Prism 5 (GraphPad Software, La Jolla, CA). The heatmap was prepared using Rstudio statistical software (Version 0.98.507–© 2009–2013 RStudio, Inc.) with the program package heatmap.2. Color codes in the heat map represent row Z-score calculated as (x–μ)/σ, where x is the measured value, μ is the mean value for all observations in the row and σ is the standard deviation of the row. Data is represented without clustering. In this figure, a negative value equal to the average size of responses towards the given antigen was assigned for a given specificity, if the patient did not mount a response towards the epitope in question in order to distinguish low frequency responses from complete absence of a response. Between-group differences of data on an interval scale were evaluated using Mann–Whitney *U*-test or students *T* test when applicable. All tests were performed two-tailed and a *P* value of 0.05 or below was considered significant. No correction for multiple comparisons was performed in this exploratory study.
