# Correction: Influence of ipilimumab on expanded tumour derived T cells from patients with metastatic melanoma

**DOI:** 10.18632/oncotarget.26359

**Published:** 2018-11-09

**Authors:** Jon Bjoern, Rikke Lyngaa, Rikke Andersen, Lisbet Rosenkrantz Hölmich, Sine Reker Hadrup, Marco Donia, Inge Marie Svane

**Affiliations:** ^1^ Center for Cancer Immune Therapy, Herlev Hospital, University of Copenhagen, Copenhagen, Denmark; ^2^ Department of Oncology, Herlev Hospital, University of Copenhagen, Copenhagen, Denmark; ^3^ Section for Immunology and Vaccinology, Technical University of Denmark, Copenhagen, Denmark; ^4^ Department of Plastic Surgery, Herlev Hospital, University of Copenhagen, Copenhagen, Denmark

**This article has been corrected:** The correct name of the 4th author is as follows:

**Lisbet Rosenkrantz Hölmich**

Original article: Oncotarget. 2017; 8:27062-27074. https://doi.org/10.18632/oncotarget.16003

